# Supplementary material for: Mechanical Properties of Ca-Saturated Hydrogels with Functionalized Alginate
Source: Gels. 2019 Apr 19;5(2):23. doi: 10.3390/gels5020023 (PMC6631140; doi:10.3390/gels5020023)
Supplement: Supplementary file 1 [file gels-05-00023-s001.zip › gels-458619-supplementary/Supplementary_file_1.pdf]

Supplementary file 1 (S1)

^{1}H NMR spectra including bulk assignment of the grafted alginates are given in Figure S1. The samples were subjected to mild acid hydrolysis as previously described [46] to reduce the viscosity prior to the NMR analysis. 6-10 mg of the samples were then dissolved in 600 µl D_2O (99.9%) and added 5 µl 3-(Trimethylsilyl)propionic 2,2,3,3-d_4 acid (TSP, Sigma Aldrich) as an internal standard and 15 µl Triethylenetetraamine-hexaacetic acid (TTHA, Sigma Aldrich) as a chelating agent for residual divalent ions. The latter was not added to samples already added EDTA (analysis of leaked alginate). 1D ^{1}H spectra were recorded at 90 °C on a Bruker Ascend 400 MHz Avance III HD spectrometer, equipped with a 5-mm SmartProbe z-gradient probe and SampleCase (Bruker BioSpin AG, Fällanden, Switzerland). The spectra were recorded using TopSpin 3.2 software (Bruker BioSpin) and processed and analyzed with TopSpin 3.5 software (Bruker BioSpin).

The degree of substitution was calculated as mole substituent per mole uronic acid residues as previously described [21,29]. The calculations are based on earlier annotations of the proton NMR spectra of alginates [32,33], periodate oxidized alginates [28], alginates grafted with methyl tyrosine ester (MeOTyr) [21], GRGDSP grafted alginates [20] and β-cyclodextrin (β-CyD) grafted alginates [29].

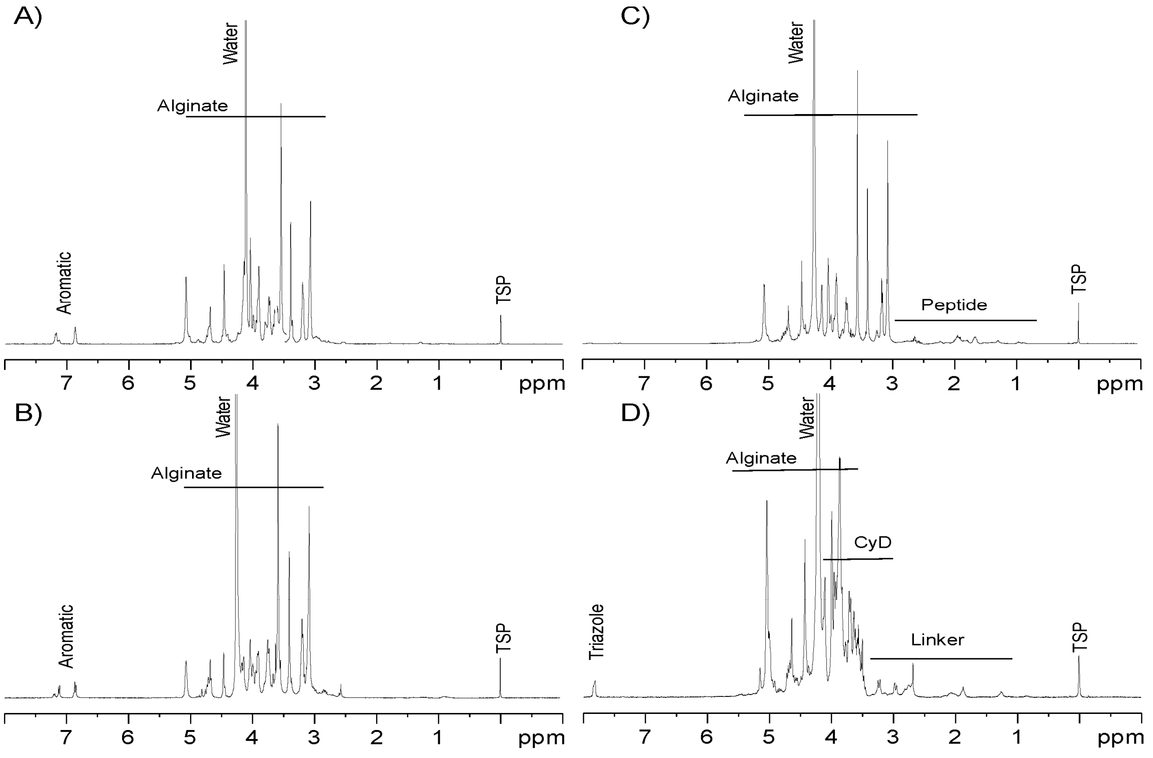

Figure S1: ^{1}H NMR of A: partially oxidized (P_0 = 0.08) *L. hyperborea* stipe alginate(65 % G) grafted with MeOTyr, B: partially oxidized (P_0 = 0.08) mannuronan grafted with MeOTyr and epimerized with AlgE64, C: partially oxidized (P_0 = 0.08) *L. hyperborea* stipe alginate grafted with GRGDSP peptide and D: partially oxidized (P_0 = 0.08) *L. hyperborea* stipe alginate grafted with β-CyD. The bulk assignment of the protons of the different chemical groups are indicated. The exact annotations of the proton NMR spectra can be found as following: alginates [32,33], periodate oxidized alginates [28], MeOTyr grafted alginates [21], GRGDSP grafted alginates [20] and β-CyD grafted alginates [29]. The 1H NMR-spectrum was recorded at 400 MHz and 90 °C in D2O.
