# Supplementary material for: Mechanical Properties of Ca-Saturated Hydrogels with Functionalized Alginate
Source: Gels. 2019 Apr 19;5(2):23. doi: 10.3390/gels5020023 (PMC6631140; doi:10.3390/gels5020023)
Supplement: Supplementary file 1 [file gels-05-00023-s001.zip › gels-458619-supplementary/Supplementary_file_2.pdf]

Supplementary file 2, S2

The molar mass of the materials was analyzed using SEC-MALS, as previously described [25]. The system consisted of a mobile phase reservoir, an on-line degasser, an HPLC isocratic pump (LC-10ADVP, Shimadzu), an autoinjector (SCL-10AV, Shimadzu), a pre-column and two serially connected columns: TSK 6000 PWXL and TSK 5000 PWXL (Toso Haas). The columns were followed by two serially connected detectors; a Dawn DSP laser light scattering photometer (Wyatt, USA) (λ_0 = 0.664 nm) and an Optilab DSP differential refractometer (Wyatt, USA). 0.15 M $NaNO_3$ with 0.01 M NaEDTA, pH 6 was used as the mobile phase. 20% acetonitrile was added to the mobile phase for the alginate grafted with β-CyD. All samples were dissolved in the mobile phase (0.25-7.5 mg/mL) and filtered (pore size 0.8 μm) prior to injection. The injection volume was adjusted to obtain an optimal light scattering signal and to avoid overloading. Astra software v. 6.1 (Wyatt, USA) was used to collect and process the obtained data, using a refractive index increment (dn/dcμ) of 0.150 mL/g for alginate samples [25].

Chromatograms and slice molecular weights for the samples are given in Figure S2.1 – S2.4.

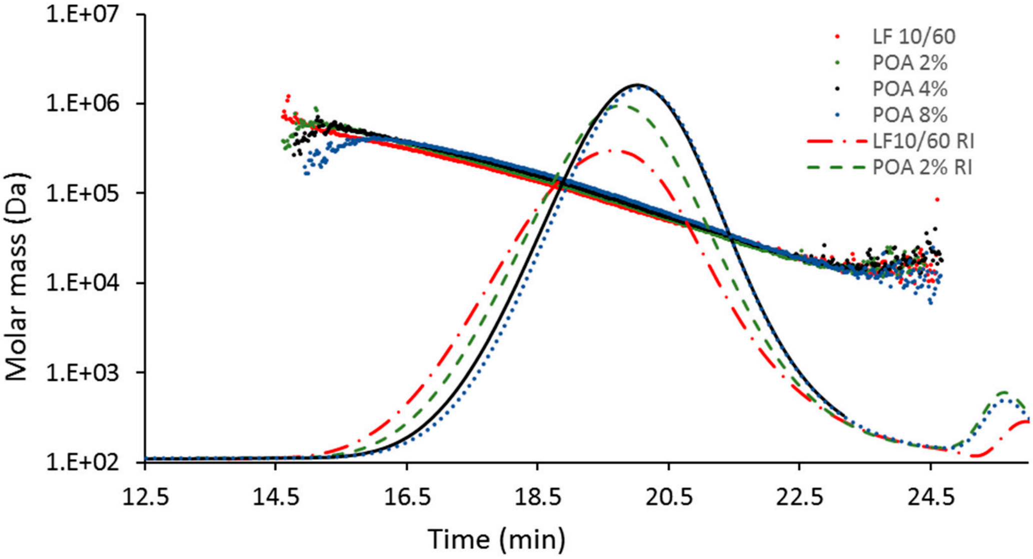

Figure S2.1: SEC-MALS data for from left to right: *Laminaria hyperborea* stipe alginate (65 %G) (red), periodate oxidized stipe alginate $P_0 = 0.02$ (green), $P_0 = 0.04$ (black), $P_0 = 0.08$ (blue). Mobile phase: 0.15 M $NaNO_3$ with 0.01 M NaEDTA, pH 6. Concentration profiles are represented with lines and slice molecular weights with dots.

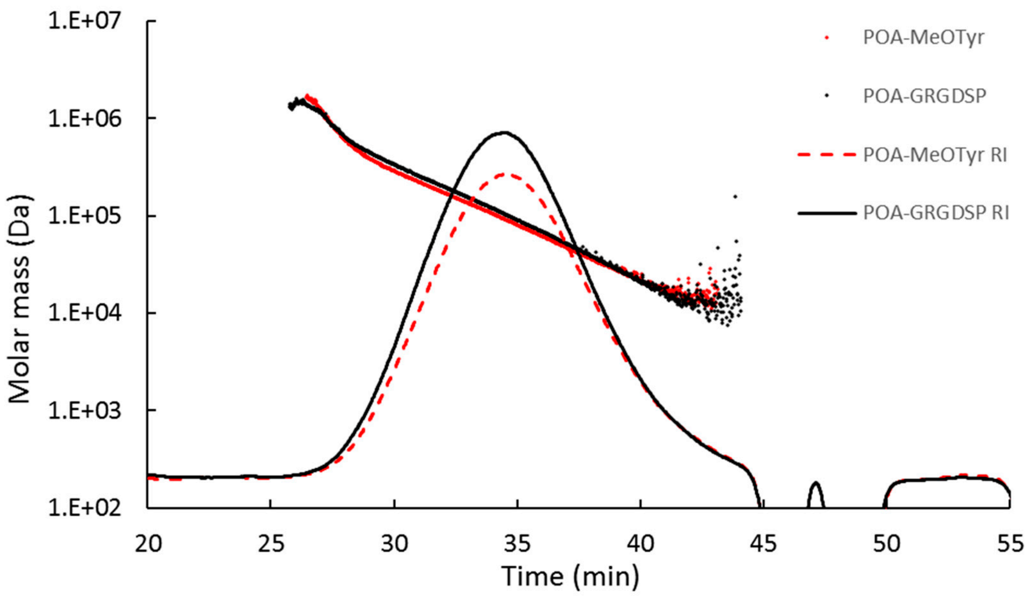

Figure S2.2: SEC-MALS data for from left to right: GRGDSP grafted stipe alginate $P_0 = 0.08$, DS = 7.0 % (POA-GRGDSP) (red) and MeOTyr grafted stipe alginate $P_0 = 0.08$, DS = 7.0 % (POA-MeOTyr) (black). Mobile phase: 0.15 M NaNO3 with 0.01 M NaEDTA, pH 6. Concentration profiles are represented with lines and slice molecular weights with dots.

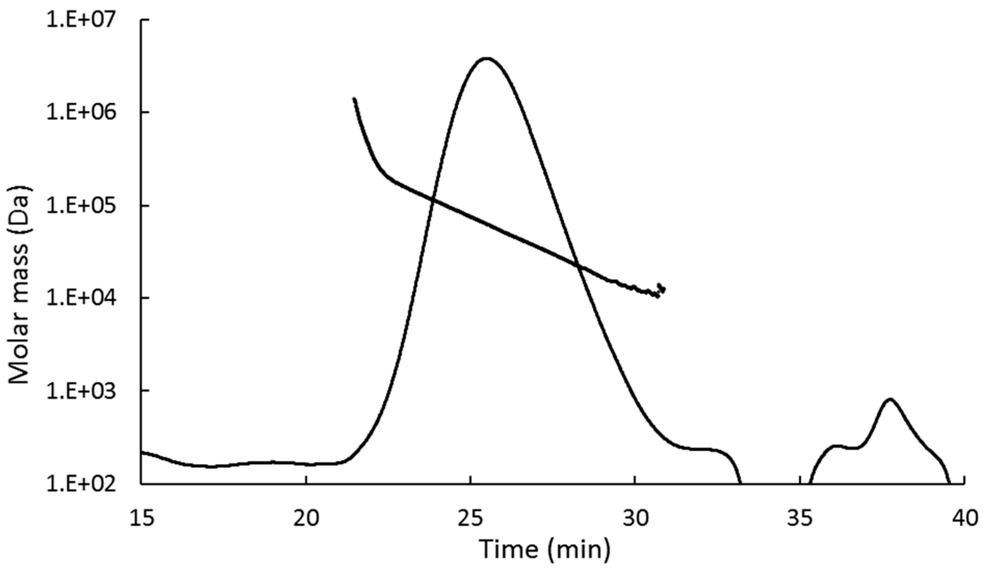

Figure S2.3: SEC-MALS data for β-CyD grafted stipe alginate $P_0 = 0.08$, DS = 1.6 % (POA-GRGDSP). Mobile phase: 0.15 M NaNO3 with 0.01 M NaEDTA, pH 6 with 20 % acetonitrile. Concentration profiles are represented with lines and slice molecular weights with dots.

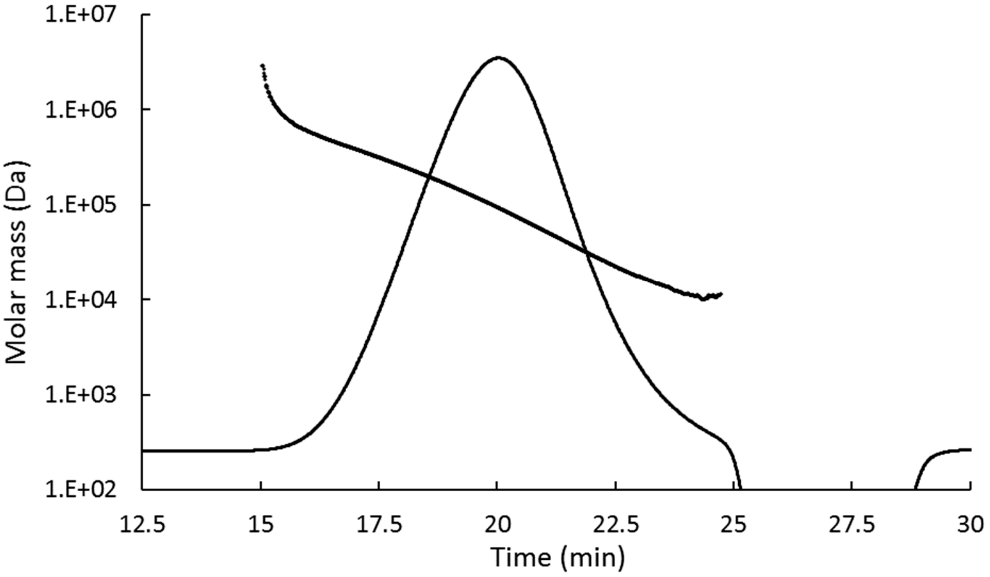

Figure S2.4: SEC-MALS data for MeOTyr grafted mannuronan, $P_0 = 0.08$, DS = 7.9 %, epimerized with AlgE64, 49 % G (epim POA-MeOTyr). Mobile phase: 0.15 M NaNO3 with 0.01 M NaEDTA, pH 6. Concentration profiles are represented with lines and slice molecular weights with dots.
