# Supplementary material for: Mechanical Properties of Ca-Saturated Hydrogels with Functionalized Alginate
Source: Gels. 2019 Apr 19;5(2):23. doi: 10.3390/gels5020023 (PMC6631140; doi:10.3390/gels5020023)
Supplement: Supplementary file 1 [file gels-05-00023-s001.zip › gels-458619-supplementary/Supplementary_file_3.pdf]

Supplementary file 3

The mechanical properties of the alginate gel cylinders were characterized by uniaxial compression using a Stable MicroSystems, TA XT plus Texture Analyser with a P/35 probe at room temperature and a compression rate of 0.1 mm/s. The weight of the loading cell was five kg. Young's modulus (E) was calculated from the initial slope (typically 0.1 – 0.3 mm) of the force/deformation curves (F/A) = E x Δl/l (see figure C1) and corrected for syneresis: $E_{corrected} = E_{measured} \times (C_{initial}/C_{final})^2$ [48]. All data were collected and processed with the "Texture Expert Exponent 32" software. Strain and stress at rupture (or ultimate compression strength) was determined in experiments where the hydrogels were compressed to the point of rupture. Reported data of stress at rupture is the maximum value on the force-deformation curve (see figure S3.1), while the deformation at rupture is reported as the compression distance at the point of rupture (mm) relative to the initial gel height (mm).

Uniaxial compression curves for the different gels are given in Figure S3.2 – S3.8.

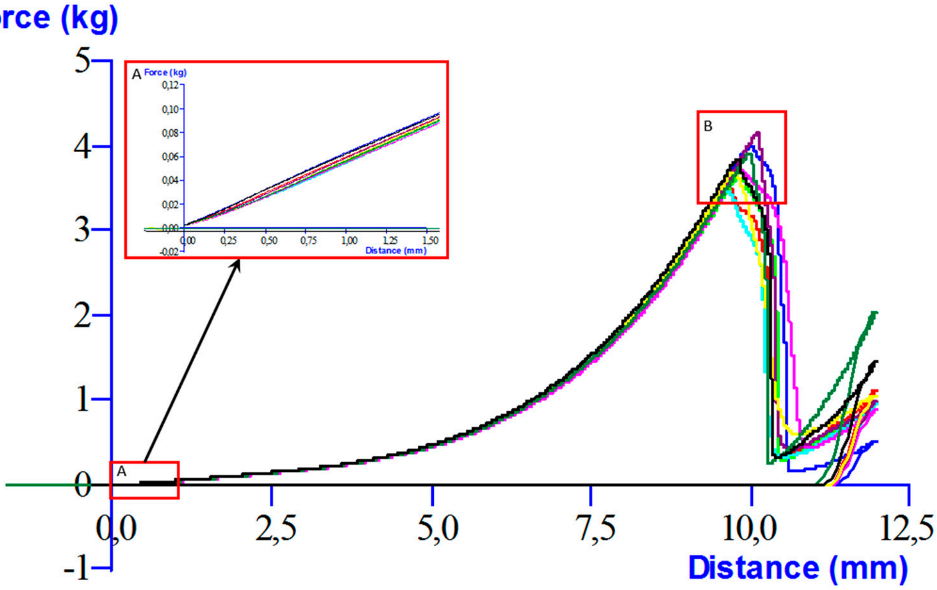

Figure S3.1: Stress (kg) – deformation (distance, mm) curves for compression of 1.0 % w/v *L. hyperborea* stipe alginate gel cylinders (n = 9). Area A shows the initial linear elastic region (typically 0.1 – 0.3 mm) used to find the initial slope from which Young's modulus is calculated. Area B shows point of rupture used to find the stress (kg) and deformation at rupture (%), calculated as the compression at rupture (mm) relative to the initial gel height (mm).

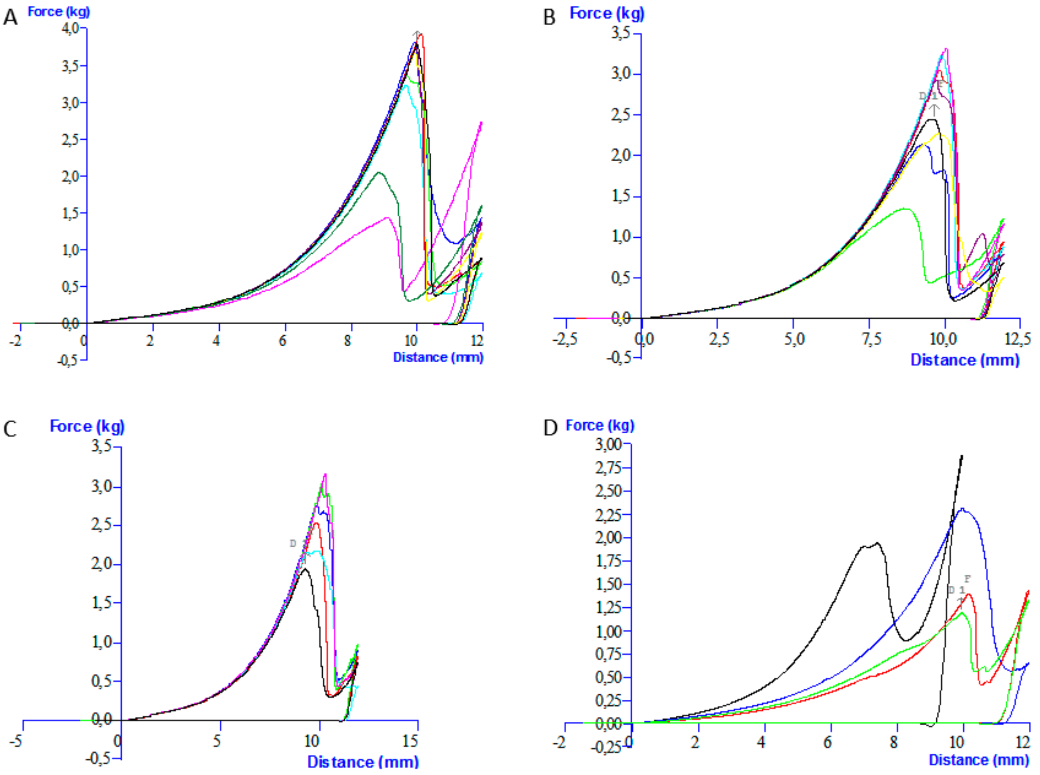

Figure S3.2: Stress (kg) – deformation (mm) curves for compression of 1.0 % w/v mixed Ca-saturated hydrogel cylinders of *L. hyperborea* stipe alginate and various fractions of POA $P_0 = 0.02$ (W_{POA}). A: $W_{POA} = 0.25$ (n= 9), B: $W_{POA} = 0.50$ (n= 9), A: $W_{POA} = 0.75$ (n= 6) and D: $W_{POA} = 1.00$ (n= 4)

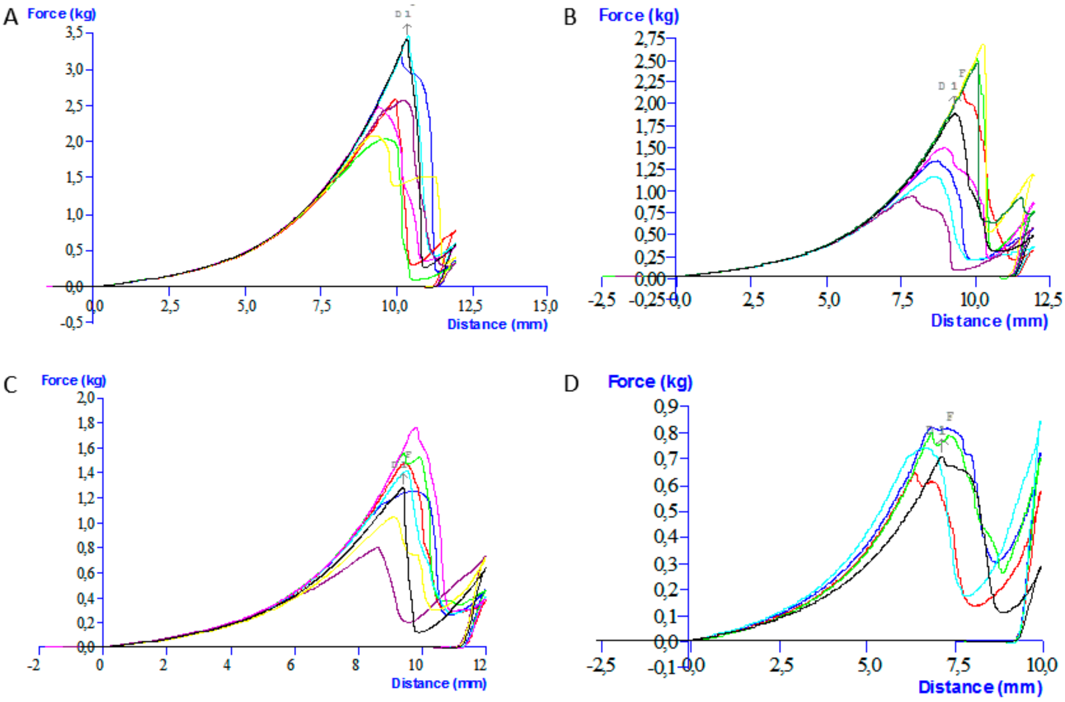

Figure S3.3: Stress (kg) – deformation (mm) curves for compression of 1.0 % w/v mixed Ca-saturated hydrogel cylinders of *L. hyperborea* stipe alginate and various fractions of POA $P_0 = 0.04$ (W_{POA}). A: $W_{POA} = 0.25$ (n= 8), B: $W_{POA} = 0.50$ (n= 9), A: $W_{POA} = 0.75$ (n= 8) and D: $W_{POA} = 1.00$ (n= 5)

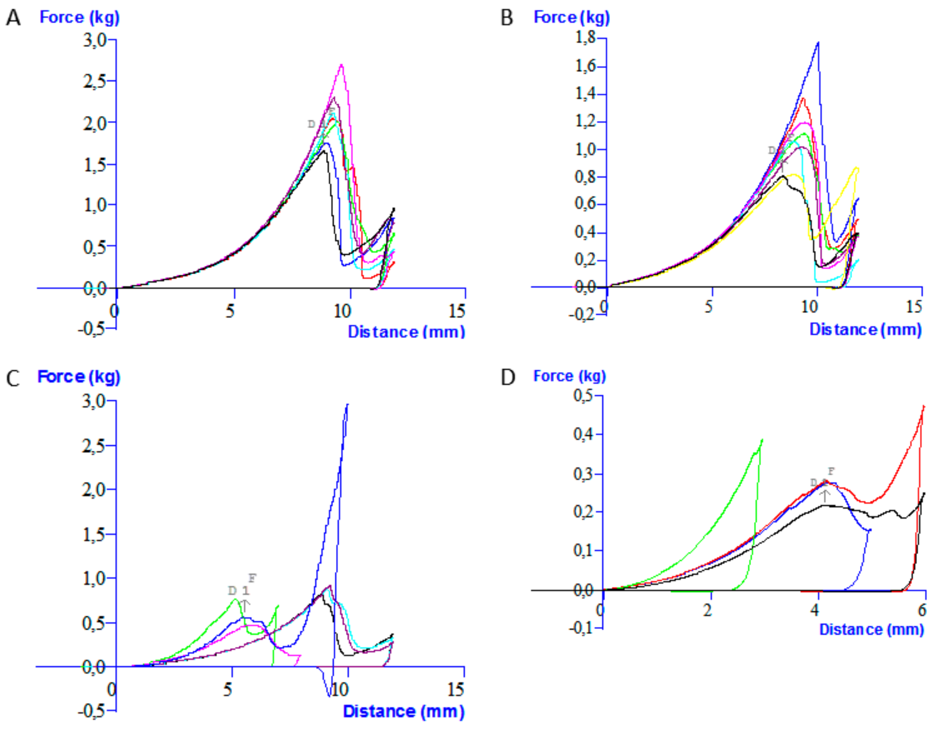

Figure S3.4: Stress (kg) – deformation (mm) curves for compression of 1.0 % w/v mixed Ca-saturated hydrogel cylinders of *L. hyperborea* stipe alginate and various fractions of POA $P_0 = 0.08$ (W_{POA}). A: $W_{POA} = 0.25$ (n= 7), B: $W_{POA} = 0.50$ (n= 8), A: $W_{POA} = 0.75$ (n= 7) and D: $W_{POA} = 1.00$ (n= 4)

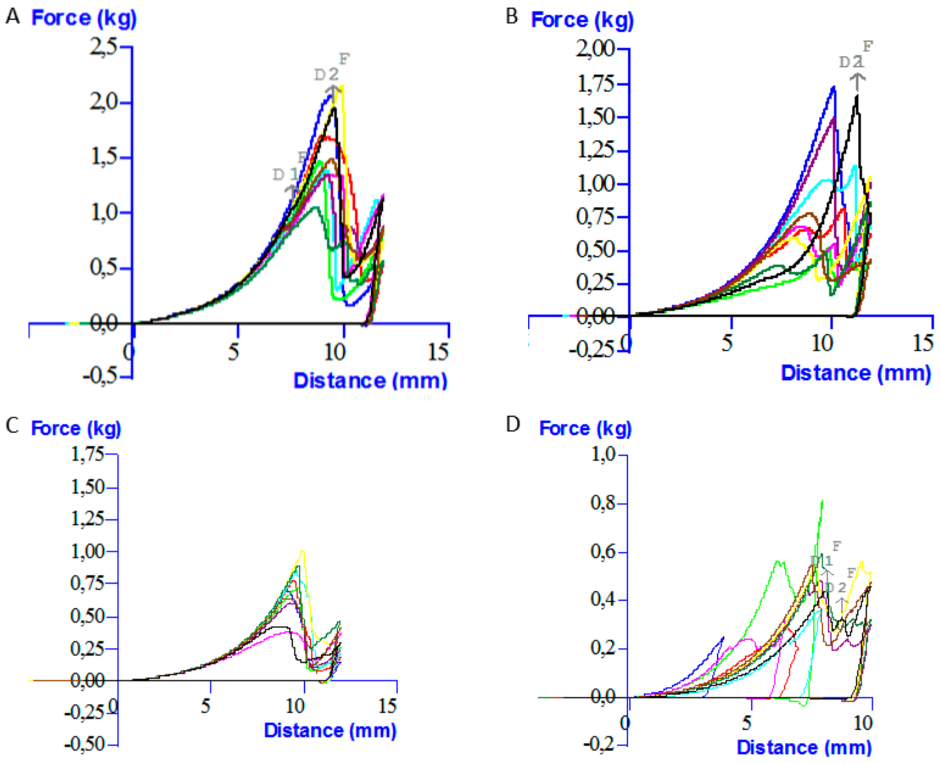

Figure S3.5: Stress (kg) – deformation (mm) curves for compression of 1.0 % w/v mixed Ca-saturated hydrogel cylinders of *L. hyperborea* stipe alginate and various fractions ($W_{POA\text{-}MeOTyr}$) of POA grafted with L-Tyrosine methyl ester (POA-MeOTyr). A: $W_{POA\text{-}MeOTyr} = 0.25$ (n= 10), B: $W_{POA\text{-}MeOTyr} = 0.50$ (n= 10), A: $W_{POA\text{-}MeOTyr} = 0.75$ (n= 10) and D: $W_{POA\text{-}MeOTyr} = 1.00$ (n= 10)

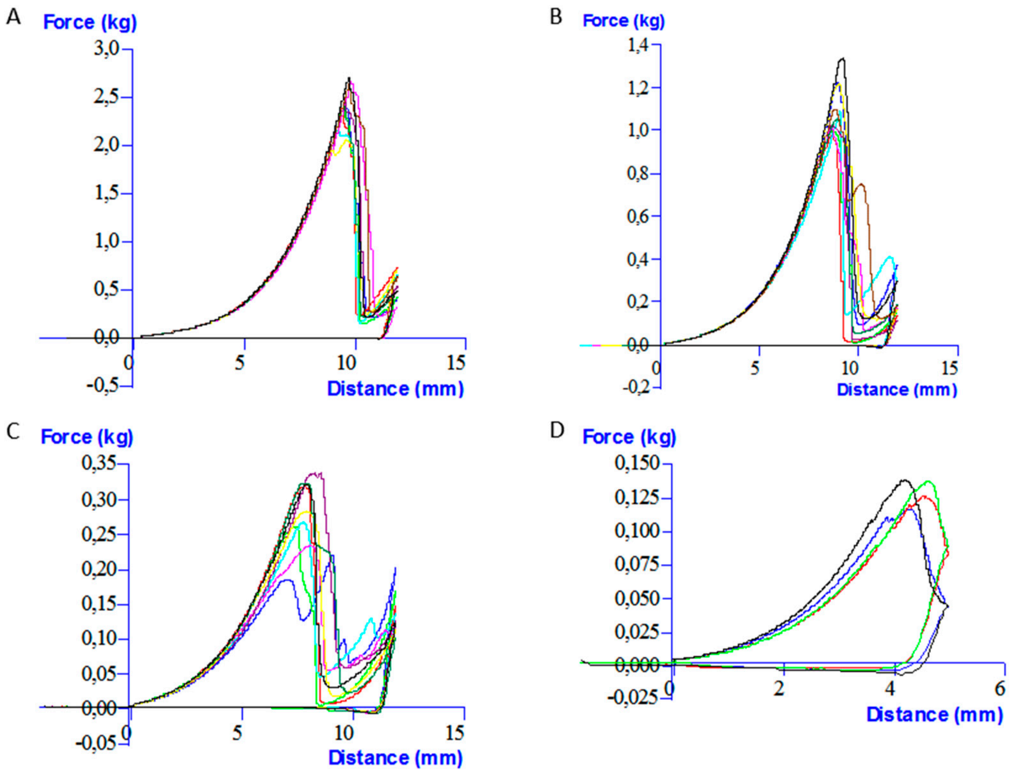

Figure S3.6: Stress (kg) – deformation (mm) curves for compression of 1.0 % w/v mixed Ca-saturated hydrogel cylinders of *L. hyperborea* stipe alginate and various fractions ($W_{POA\text{-}\beta CyD}$) of POA grafted with β-cyclodextrin (POA-βCyD). A: $W_{POA\text{-}\beta CyD}$ = 0.25 (n= 10), B: $W_{POA\text{-}\beta CyD}$= 0.50 (n= 10), A: $W_{POA\text{-}\beta CyD}$ = 0.75 (n= 9) and D: $W_{POA\text{-}\beta CyD}$ = 1.00 (n= 4)

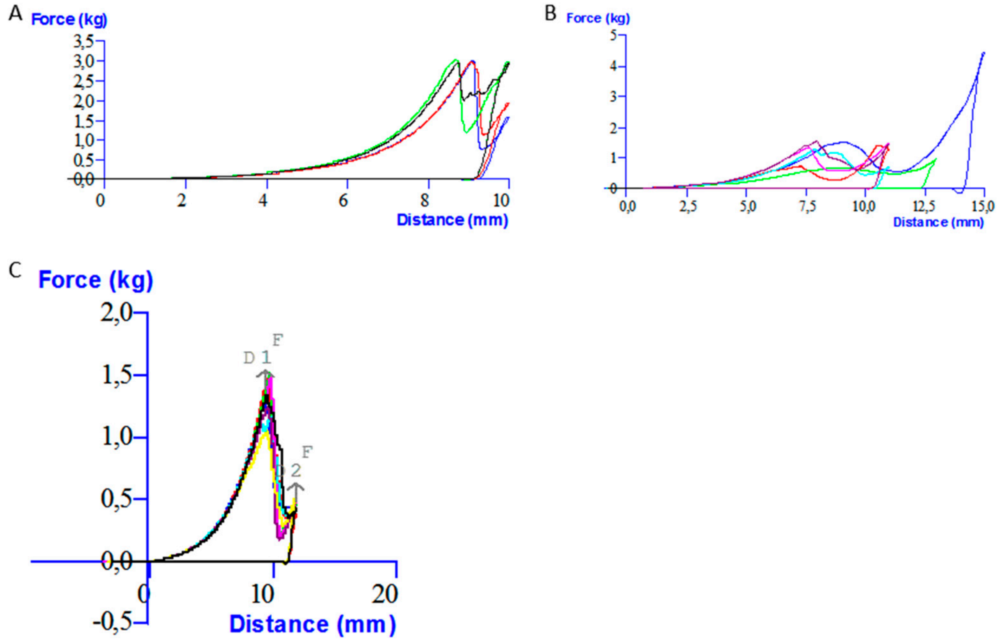

Figure S3.7: Stress (kg) – deformation (mm) curves for compression of 1.0 % w/v mixed Ca-saturated hydrogel cylinders of A: *L. hyperborea* stipe alginate and EpimPOA-MeOTyr (epimerized mannuronan grafted with MeOTyr) $W_{EpimPOA\text{-}MeOTyr}$ = 0.50 (n = 5), B: *L. hyperborea* stipe alginate and EpimPOA-MeOTyr, $W_{EpimPOA\text{-}MeOTyr}$ = 1.00 (n = 4) and C: *L. hyperborea* stipe alginate and POA grafted with GRGDSP (POA-GRGDSP), $W_{POA\text{-}GRGDSP}$ = 0.50 (n = 8).

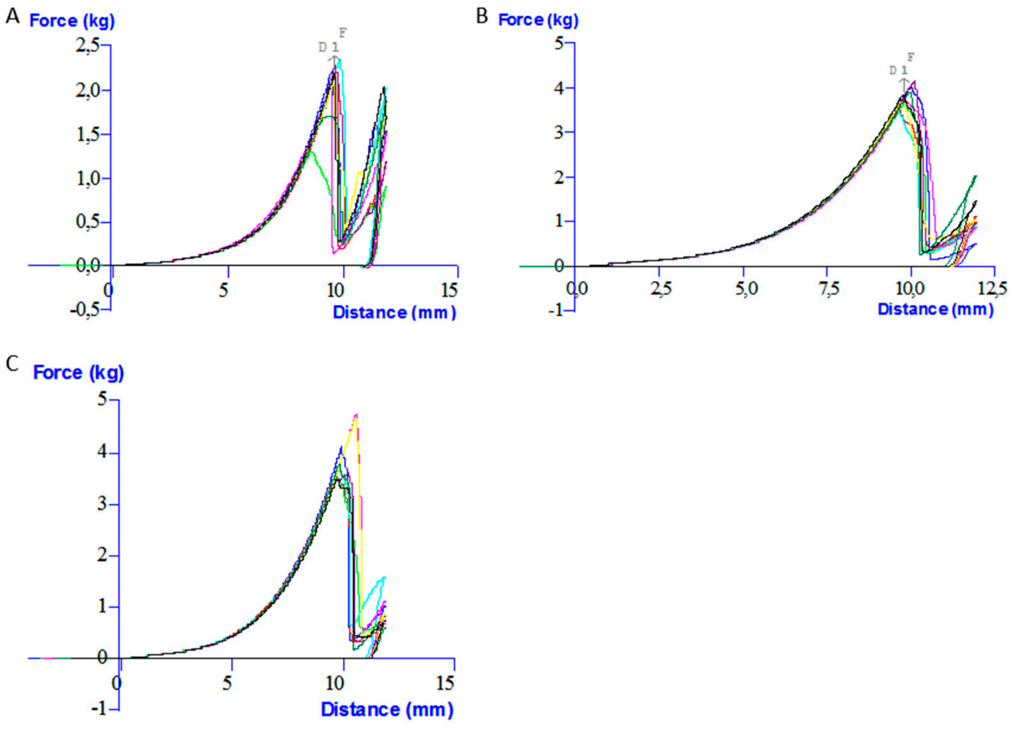

Figure S3.8: Stress (kg) – deformation (mm) curves for compression of A: 0.5 % w/v *L. hyperborea* stipe alginate gel cylinders (n = 9), B: 1.0 % w/v *L. hyperborea* stipe alginate gel cylinders (n = 9) and C: 1.0 % w/v *L. hyperborea* stipe alginate gel cylinders with free β-CyD (n = 9).
