# Supplementary material for: Mechanical Properties of Ca-Saturated Hydrogels with Functionalized Alginate
Source: Gels. 2019 Apr 19;5(2):23. doi: 10.3390/gels5020023 (PMC6631140; doi:10.3390/gels5020023)
Supplement: Supplementary file 1 [file gels-05-00023-s001.zip › gels-458619-supplementary/Supplementary_file_4.pdf]

Supplementary file 4

Statistical data for the modified alginate gels are here given with regards to the Young's modulus, syneresis and compression and stress at rupture.

Table S4.1. Statistical data of the partially oxidized alginate gels with regards to the pure stipe alginate 1% gels. The values for Young's modulus and syneresis are given as the mean of the gels. Abbreviations: n = number of gels. SD = standard deviation. POA = partially oxidized alginate.

Oxidation degree (%)	Fraction of POA	Young's modulus (kPa)	SD	n	p-value	Syneresis (%)	SD	n	p-value
2	0.25	28.4	2.9	9	0.50	30.4	1.4	9	1.0
	0.50	28.1	0.6	8	0.41	27.1	0.6	8	<0.01
	0.75	24.9	2.8	6	<0.01	27.3	0.8	6	0.059
	1.00	8.7	5.4	5	<0.01	28.1	3.1	3	0.84
4	0.25	32.2	1.7	8	1.00	16.7	0.8	8	<0.01
	0.50	26.1	1.4	8	<0.01	21.1	0.8	8	<0.01
	0.75	19.6	2.6	7	<0.01	19.8	0.8	7	<0.01
	1.00	8.9	1.8	5	<0.01	39.2	3.6	5	<0.01
8	0.25	29.3	1.3	6	0.96	23.0	0.6	6	<0.01
	0.50	23.8	2.4	8	<0.01	15.2	0.8	8	<0.01
	0.75	7.1	5.2	7	<0.01	17.7	1.1	3	<0.01

Table S4.2. Statistical data of the partially oxidized alginate gels with regards to the pure stipe alginate 1% gels. The values for deformation at rupture (compression) and stress at rupture are given as the mean of the gels. Abbreviations: n = number of gels. SD = standard deviation. POA = partially oxidized alginate.

**Oxidation degree (%)**	**Fraction of POA**	**Deformation (%)**	**SD**	**n**	**p-value**	**Stress at rupture (N)**	**SD**	**n**	**p-value**
**2**	0.25	62.2	2.0	7	1.0	35.8	2.5	7	1.0
	0.50	59	1.8	7	0.94	27.3	4.7	7	<0.01
	0.75	59.4	2.4	6	0.99	25.5	4.7	6	<0.01
	1.00	61.3	3.4	5	1.0	15.1	5.5	5	<0.01
**4**	0.25	58.4	2.7	8	0.73	26.8	5.5	8	<0.01
	0.50	55.7	5.2	8	0.017	17.3	6.3	8	<0.01
	0.75	56.9	1.0	6	0.21	14.3	1.8	6	<0.01
	1.00	53.2	1.8	5	<0.01	7.3	0.7	5	<0.01
**8**	0.25	57.9	2.4	6	0.60	21.3	3.2	6	<0.01
	0.50	53.9	3.1	7	<0.01	11.3	3.3	7	<0.01
	0.75	53.3	3.9	7	<0.01	7.1	1.7	7	<0.01

Table S4.3. Statistical data of the partially oxidized alginate gels with regards to the pure stipe alginate 0.5% gels. The values for Young's modulus and syneresis are given as the mean

of the gels. Abbreviations: n = number of gels. SD = standard deviation. POA = partially oxidized alginate.

Oxidation degree (%)	Fraction of POA	Young's modulus (kPa)	SD	n	p-value	Syneresis (%)	SD	n	p-value
**2**	0.25	28.4	2.9	9	<0.01	30.4	1.4	9	<0.01
	0.50	28.1	0.6	8	<0.01	27.1	0.6	8	<0.01
	0.75	24.9	2.8	6	<0.01	27.3	0.8	6	<0.01
	1.00	8.7	5.4	5	0.92	28.1	3.1	3	<0.01
**4**	0.25	32.2	1.7	8	<0.01	16.7	0.8	8	<0.01
	0.50	26.1	1.4	8	<0.01	21.1	0.8	8	<0.01
	0.75	19.6	2.6	7	<0.01	19.8	0.8	7	<0.01
	1.00	8.9	1.8	5	0.89	39.2	3.6	5	<0.01
**8**	0.25	29.3	1.3	6	<0.01	23.0	0.6	6	<0.01
	0.50	23.8	2.4	8	<0.01	15.2	0.8	8	<0.01
	0.75	7.1	5.2	7	1.0	17.7	1.1	3	<0.01

Table S.4.4. Statistical data of the partially oxidized alginate gels with regards to the pure stipe alginate 0.5% gels. The values for deformation at rupture (compression) and stress at rupture are given as the mean of the gels. Abbreviations: n = number of gels. SD = standard deviation. POA = partially oxidized alginate.

Oxidation degree (%)	Fraction of POA	Deformation (%)	SD	n	p-value	Stress at rupture (N)	SD	n	p-value
**2**	0.25	62.2	2.0	7	0.047	35.8	2.5	7	<0.01
	0.50	59	1.8	7	<0.01	27.3	4.7	7	0.021
	0.75	59.4	2.4	6	<0.01	25.5	4.7	6	0.28
	1.00	61.3	3.4	5	0.028	15.1	5.5	5	0.55
**4**	0.25	58.4	2.7	8	<0.01	26.8	5.5	8	0.031
	0.50	55.7	5.2	8	<0.01	17.3	6.3	8	0.96
	0.75	56.9	1.0	6	<0.01	14.3	1.8	6	0.23
	1.00	53.2	1.8	5	<0.01	7.3	0.7	5	<0.01
**8**	0.25	57.9	2.4	6	<0.01	21.3	3.2	6	1.0
	0.50	53.9	3.1	7	<0.01	11.3	3.3	7	<0.01
	0.75	53.3	3.9	7	<0.01	7.1	1.7	7	<0.01

Table S4.5. Statistical data comparing the partially oxidized alginate gels with regards to the oxidation degree. Data for the 1% and 0.5% pure stipe alginate gels are also given. The values for Young's modulus are given as the mean of the gels. Abbreviations: n = number of gels. SD = standard deviation. POA 0.02 = 2% partially oxidized alginate. POA 0.04 = 4% partially oxidized alginate. POA 0.08 = 8% partially oxidized alginate.

Fraction of POA	Gel A / Gel B	Young's modulus (kPa)	SD	n	p-value
**0**	1% gel / 0.5% gel	31.4 / 6.3	2.7 / 1.0	8 / 9	<0.01
**0.25**	POA 0.02 / POA 0.04	28.4 / 32.2	2.9 / 1.7	9 / 8	0.17

	POA 0.02 / POA 0.08	28.4 / 29.3	2.9 / 1.3	9 / 6	1.0
	POA 0.04 / POA 0.08	32.3 / 29.3	1.7 / 1.3	8 / 6	0.72
**0.50**	POA 0.02 / POA 0.04	28.1 / 26.1	0.6 / 1.4	8 / 8	0.95
	POA 0.02 / POA 0.08	28.1 / 23.8	0.6 / 2.4	8 / 8	0.084
	POA 0.04 / POA 0.08	26.1 / 23.8	1.4 / 2.4	8 / 8	0.88
**0.75**	POA 0.02 / POA 0.04	24.9 / 19.6	2.8 / 2.6	6 / 7	0.032
	POA 0.02 / POA 0.08	24.9 / 7.1	2.8 / 5.2	6 / 7	<0.01
	POA 0.04 / POA 0.08	19.6 / 7.1	2.6 / 5.2	7 / 7	<0.01
**1.00**	POA 0.02 / POA 0.04	8.7 / 8.9	5.4 / 1.8	5 / 5	1.0

Table S4.6. Statistical data comparing the partially oxidized alginate gels with regards to the oxidation degree. Data for the 1% and 0.5% pure stipe alginate gels are also given. The values for syneresis are given as the mean of the gels. Abbreviations: n = number of gels. SD = standard deviation. POA 0.02 = 2% partially oxidized alginate. POA 0.04 = 4% partially oxidized alginate. POA 0.08 = 8% partially oxidized alginate.

**Fraction of POA**	**Gel A / Gel B**	**Syneresis (%)**	**SD**	**n**	**p-value**
**0**	1% gel / 0.5% gel	29.7 / 45.7	0.7 / 1.7	9 / 9	<0.01
**0.25**	POA 0.02 / POA 0.04	30.4 / 16.7	1.4 / 0.8	9 / 8	<0.01
	POA 0.02 / POA 0.08	30.4 / 23.0	1.4 / 0.6	9 / 6	<0.01
	POA 0.04 / POA 0.08	16.7 / 23.0	0.8 / 0.6	8 / 6	<0.01
**0.50**	POA 0.02 / POA 0.04	27.1 / 21.1	0.6 / 0.8	8 / 8	<0.01
	POA 0.02 / POA 0.08	27.1 / 15.2	0.6 / 0.8	8 / 8	<0.01
	POA 0.04 / POA 0.08	21.1 / 15.2	0.8 / 0.8	8 / 8	<0.01
**0.75**	POA 0.02 / POA 0.04	27.3 / 19.8	0.8 / 0.8	6 / 7	<0.01
	POA 0.02 / POA 0.08	27.3 / 17.7	0.8 / 1.1	6 / 3	<0.01
	POA 0.04 / POA 0.08	19.8 / 17.7	0.8 / 1.1	7 / 3	0.58
**1.00**	POA 0.02 / POA 0.04	NA	NA	NA	NA

Table S4.7. Statistical data comparing the partially oxidized alginate gels with regards to the oxidation degree. Data for the 1% and 0.5% pure stipe alginate gels are also given. The values for deformation at rupture (compression) are given as the mean of the gels. Abbreviations: n = number of gels. SD = standard deviation. POA 0.02 = 2% partially oxidized alginate. POA 0.04 = 4% partially oxidized alginate. POA 0.08 = 8% partially oxidized alginate.

**Fraction of POA**	**Gel A / Gel B**	**Deformation (%)**	**SD**	**n**	**p-value**
**0**	1% gel / 0.5% gel	61.4 / 67.4	1.1 / 3.6	8 / 9	<0.01
**0.25**	POA 0.02 / POA 0.04	62.2 / 58.4	2.0 / 2.7	7 / 8	0.43
	POA 0.02 / POA 0.08	62.2 / 57.9	2.0 / 2.4	7 / 6	0.32
	POA 0.04 / POA 0.08	58.4 / 57.9	2.7 / 2.4	8 / 6	1.0
**0.50**	POA 0.02 / POA 0.04	59.0 / 55.7	1.8 / 5.2	7 / 8	0.64
	POA 0.02 / POA 0.08	59.0 / 53.9	1.8 / 3.1	7 / 7	0.088
	POA 0.04 / POA 0.08	55.7 / 53.9	5.2 / 3.1	8 / 7	1.0
**0.75**	POA 0.02 / POA 0.04	59.4 / 56.9	2.4 / 1.0	6 / 6	0.96

	POA 0.02 / POA 0.08	59.4 / 53.3	2.4 / 3.9	6 / 7	0.026
	POA 0.04 / POA 0.08	56.9 / 53.3	1.8 / 3.9	6 / 7	0.66
**1.00**	POA 0.02 / POA 0.04	61.3 / 53.2	3.4 / 1.8	5 / 5	<0.01

Table S4.8. Statistical data comparing the partially oxidized alginate gels with regards to the oxidation degree. Data for the 1% and 0.5% pure stipe alginate gels are also given. The values for stress at rupture are given as the mean of the gels. Abbreviations: n = number of gels. SD = standard deviation. POA 0.02 = 2% partially oxidized alginate. POA 0.04 = 4% partially oxidized alginate. POA 0.08 = 8% partially oxidized alginate.

Fraction of POA	Gel A / Gel B	Stress at rupture (N)	SD	n	p-value
**0**	1% gel / 0.5% gel	37.1 / 20.0	2.4 / 3.3	8 / 9	<0.01
**0.25**	POA 0.02 / POA 0.04	35.8 / 26.8	2.5 / 5.5	7 / 8	<0.01
	POA 0.02 / POA 0.08	35.8 / 21.3	2.5 / 3.2	7 / 6	<0.01
	POA 0.04 / POA 0.08	26.8 / 21.3	5.5 / 3.2	8 / 6	0.30
**0.50**	POA 0.02 / POA 0.04	27.3 / 17.3	4.7 / 6.3	7 / 8	<0.01
	POA 0.02 / POA 0.08	27.3 / 11.3	4.7 / 3.3	7 / 7	<0.01
	POA 0.04 / POA 0.08	17.3 / 11.3	6.3 / 3.3	8 / 7	0.16
**0.75**	POA 0.02 / POA 0.04	25.5 / 14.3	4.7 / 1.8	6 / 6	<0.01
	POA 0.02 / POA 0.08	25.5 / 7.1	4.7 / 1.7	6 / 7	<0.01
	POA 0.04 / POA 0.08	14.3 / 7.1	1.8 / 1.7	6 / 7	0.061
**1.00**	POA 0.02 / POA 0.04	15.1 / 7.3	5.5 / 0.7	5 / 5	0.093

Table S4.9. Statistical data comparing the partially oxidized alginate gels with regards to the fraction of modified alginate in the gels. The values for Young's modulus are given as the mean of the gels. Abbreviations: n = number of gels. SD = standard deviation. POA = partially oxidized alginate.

Oxidation degree (%)	Fraction of POA	Young's modulus (kPa)	SD	n	p-value
**2**	0.25 / 0.50	28.4 / 28.1	2.9 / 0.6	9 / 8	1.0
	0.25 / 0.75	28.4 / 24.9	2.9 / 2.8	9 / 6	0.40
	0.25 / 1.0	28.4 / 8.7	2.9 / 5.4	9 / 5	<0.01
	0.50 / 0.75	28.1 / 24.9	0.6 / 2.8	8 / 6	0.57
	0.50 / 1.0	28.1 / 8.7	0.6 / 5.4	8 / 5	<0.01
	0.75 / 1.0	24.9 / 8.7	2.8 / 5.4	6 / 5	<0.01
**4**	0.25 / 0.50	32.2 / 26.1	1.7 / 1.4	8 / 8	<0.01
	0.25 / 0.75	32.2 / 19.6	1.7 / 2.6	8 / 7	<0.01
	0.25 / 1.0	32.2 / 8.9	1.7 / 1.8	8 / 5	<0.01
	0.50 / 0.75	26.1 / 19.6	1.4 / 2.6	8 / 7	<0.01
	0.50 / 1.0	26.1 / 8.9	1.4 / 1.8	8 / 5	<0.01
	0.75 / 1.0	19.6 / 8.9	2.6 / 1.8	7 / 5	<0.01
**8**	0.25 / 0.50	29.3 / 23.8	1.3 / 2.4	6 / 8	0.02
	0.25 / 0.75	29.3 / 7.1	1.3 / 5.2	6 / 7	<0.01
	0.50 / 0.75	23.8 / 7.1	2.4 / 5.2	8 / 7	<0.01

Table S4.10. Statistical data comparing the partially oxidized alginate gels with regards to the fraction of modified alginate in the gels. The values for syneresis are given as the mean of the gels. Abbreviations: n = number of gels. SD = standard deviation. POA = partially oxidized alginate.

Oxidation degree (%)	Fraction of POA	Syneresis (%)	SD	n	p-value
2	0.25 / 0.50	30.4 / 27.1	1.4 / 0.6	9 / 8	<0.01
	0.25 / 0.75	30.4 / 27.3	1.4 / 0.8	9 / 6	<0.01
	0.25 / 1.0	30.4 / 28.1	1.4 / 3.1	9 / 3	0.34
	0.50 / 0.75	27.1 / 27.3	0.6 / 0.8	8 / 6	1.0
	0.50 / 1.0	27.1 / 28.1	0.6 / 3.1	8 / 3	1.0
	0.75 / 1.0	27.3 / 28.1	0.8 / 3.1	6 / 3	1.0
4	0.25 / 0.50	16.7 / 21.4	0.8 / 0.8	8 / 8	<0.01
	0.25 / 0.75	16.7 / 19.8	0.8 / 0.8	8 / 7	<0.01
	0.25 / 1.0	16.7 / 39.2	0.8 / 3.6	8 / 5	<0.01
	0.50 / 0.75	21.4 / 19.8	0.8 / 0.8	8 / 7	0.81
	0.50 / 1.0	21.4 / 39.2	0.8 / 3.6	8 / 5	<0.01
	0.75 / 1.0	19.8 / 39.2	0.8 / 3.6	7 / 5	<0.01
8	0.25 / 0.50	23.0 / 15.2	0.6 / 0.8	6 / 8	<0.01
	0.25 / 0.75	23.0 / 17.7	0.6 / 1.1	6 / 3	<0.01
	0.50 / 0.75	15.2 / 17.7	0.8 / 1.1	8 / 3	0.28

Table S4.11. Statistical data comparing the partially oxidized alginate gels with regards to the fraction of modified alginate in the gels. The values for deformation at rupture (compression) are given as the mean of the gels. Abbreviations: n = number of gels. SD = standard deviation. POA = partially oxidized alginate.

Oxidation degree (%)	Fraction of POA	Deformation (%)	SD	n	p-value
2	0.25 / 0.50	62.2 / 59.0	2.0 / 1.8	7 / 7	0.73
	0.25 / 0.75	62.2 / 59.4	2.0 / 2.4	7 / 6	0.89
	0.25 / 1.0	62.2 / 61.3	2.0 / 3.4	7 / 5	1.0
	0.50 / 0.75	59.0 / 59.4	1.8 / 2.4	7 / 6	1.0
	0.50 / 1.0	59.0 / 61.3	1.8 / 3.4	7 / 5	0.98
	0.75 / 1.0	59.4 / 61.3	2.4 / 3.4	6 / 5	1.0
4	0.25 / 0.50	58.4 / 55.7	2.7 / 5.2	8 / 8	0.94
	0.25 / 0.75	58.4 / 56.9	2.7 / 1.0	8 / 6	1.0
	0.25 / 1.0	58.4 / 53.2	2.7 / 1.8	8 / 5	0.12
	0.50 / 0.75	55.7 / 56.9	5.2 / 1.0	8 / 6	1.0
	0.50 / 1.0	55.7 / 53.2	5.2 / 1.8	8 / 5	0.95
	0.75 / 1.0	56.9 / 53.2	1.0 / 1.8	6 / 5	0.72
8	0.25 / 0.50	57.9 / 53.9	2.4 / 3.1	6 / 7	0.46
	0.25 / 0.75	57.9 / 53.3	2.4 / 3.9	6 / 7	0.26
	0.50 / 0.75	53.9 / 53.3	3.1 / 3.9	7 / 7	1.0

Table S4.12. Statistical data comparing the partially oxidized alginate gels with regards to the fraction of modified alginate in the gels. The values for stress at rupture are given as the

mean of the gels. Abbreviations: n = number of gels. SD = standard deviation. POA = partially oxidized alginate.

Oxidation degree (%)	Fraction of POA	Stress at rupture (N)	SD	n	p-value
2	0.25 / 0.50	35.8 / 27.3	2.5 / 4.7	7 / 7	<0.01
	0.25 / 0.75	35.8 / 25.5	2.5 / 4.7	7 / 6	<0.01
	0.25 / 1.0	35.8 / 15.1	2.5 / 5.5	7 / 5	<0.01
	0.50 / 0.75	27.3 / 25.5	4.7 / 4.7	7 / 6	1.0
	0.50 / 1.0	27.3 / 15.1	4.7 / 5.5	7 / 5	<0.01
	0.75 / 1.0	25.5 / 15.1	4.7 / 5.5	6 / 5	<0.01
4	0.25 / 0.50	26.8 / 17.3	5.5 / 6.3	8 / 8	<0.01
	0.25 / 0.75	26.8 / 14.3	5.5 / 1.8	8 / 6	<0.01
	0.25 / 1.0	26.8 / 7.3	5.5 / 0.7	8 / 5	<0.01
	0.50 / 0.75	17.3 / 14.3	6.3 / 1.8	8 / 6	0.97
	0.50 / 1.0	17.3 / 7.3	6.3 / 0.7	8 / 5	<0.01
	0.75 / 1.0	14.3 / 7.3	1.8 / 0.7	6 / 5	0.15
8	0.25 / 0.50	21.3 / 11.3	3.2 / 3.3	6 / 7	<0.01
	0.25 / 0.75	21.3 / 7.1	3.2 / 1.7	6 / 7	<0.01
	0.50 / 0.75	11.3 / 7.1	3.3 / 1.7	7 / 7	0.70

Table S4.13. Statistical data of the partially oxidized alginate gels with regards to the pure stipe alginate 1% gels. The values for Young's modulus are given as the mean of the gels. Abbreviations: n = number of gels. SD = standard deviation. POA = partially oxidized alginate. βCyD = β-cyclodextrin. POA-MeOTyr = POA grafted with L-Tyrosine methyl ester. POA-βCyD = POA grafted with β-cyclodextrin. POA-GRGDSP = POA grafted with peptide GRGDSP.

Unmodified alginate mixed with:	Fraction of modified alginate	Young's modulus (kPa)	SD	n	p-value
**free βCyD**	NA	30.4	2.3	9	1.0
**POA-MeOTyr**	0.25	21.1	2.4	10	<0.01
	0.50	15.0	2.1	10	<0.01
	0.75	7.7	0.4	10	<0.01
**POA-βCyD**	0.25	21.9	4.4	10	<0.01
	0.50	14.5	1.2	10	<0.01
	0.75	6.7	1.2	9	<0.01
**POA-GRGDSP**	0.50	14.0	0.9	8	<0.01

Table S4.14. Statistical data of the partially oxidized alginate gels with regards to the pure stipe alginate 1% gels. The values for syneresis are given as the mean of the gels. Abbreviations: n = number of gels. SD = standard deviation. POA = partially oxidized alginate. βCyD = β-cyclodextrin. POA-MeOTyr = POA grafted with L-Tyrosine methyl ester. POA-βCyD = POA grafted with β-cyclodextrin. POA-GRGDSP = POA grafted with peptide GRGDSP.

Unmodified alginate mixed with:	Fraction of modified alginate	Syneresis (%)	SD	n	p-value
**free βCyD**	NA	25.0	1.4	9	<0.01
**POA-MeOTyr**	0.25	25.4	0.5	10	<0.01
	0.50	22.9	2.6	10	<0.01
	0.75	20.9	0.8	10	<0.01
**POA-βCyD**	0.25	19.5	1.1	10	<0.01
	0.50	15.2	1.0	10	<0.01
	0.75	15.6	2.1	9	<0.01
**POA-GRGDSP**	0.50	19.0	1.8	8	<0.01

Table S4.15. Statistical data of the partially oxidized alginate gels with regards to the pure stipe alginate 1% gels. The values for deformation at rupture (compression) are given as the mean of the gels. Abbreviations: n = number of gels. SD = standard deviation. POA = partially oxidized alginate. βCyD = β-cyclodextrin. POA-MeOTyr = POA grafted with L-Tyrosine methyl ester. POA-βCyD = POA grafted with β-cyclodextrin. POA-GRGDSP = POA grafted with peptide GRGDSP.

Unmodified alginate mixed with:	Fraction of modified alginate	Deformation (%)	SD	n	p-value
**free βCyD**	NA	64.6	2.9	9	0.87
**POA-MeOTyr**	0.25	58.0	2.3	10	0.78
	0.50	61.6	6.8	10	1.0
	0.75	58.3	2.5	10	0.88
**POA-βCyD**	0.25	61.3	7.1	10	1.0
	0.50	54.0	1.8	10	<0.01
	0.75	48.6	3.3	9	<0.01
**POA-GRGDSP**	0.50	58.0	2.1	8	0.85

Table S4.16. Statistical data of the partially oxidized alginate gels with regards to the pure stipe alginate 1% gels. The values for the stress at rupture are given as the mean of the gels. Abbreviations: n = number of gels. SD = standard deviation. POA = partially oxidized alginate. βCyD = β-cyclodextrin. POA-MeOTyr = POA grafted with L-Tyrosine methyl ester. POA-βCyD = POA grafted with β-cyclodextrin. POA-GRGDSP = POA grafted with peptide GRGDSP.

Unmodified alginate mixed with:	Fraction of modified alginate	Stress at rupture (N)	SD	n	p-value
**free βCyD**	NA	38.4	4.9	9	1.0
**POA-MeOTyr**	0.25	15.8	3.6	10	<0.01
	0.50	9.9	4.6	10	<0.01
	0.75	6.9	1.9	10	<0.01
**POA-βCyD**	0.25	24.0	2.0	10	<0.01
	0.50	10.9	1.2	10	<0.01
	0.75	2.8	0.4	9	<0.01
**POA-GRGDSP**	0.50	13.0	1.8	8	<0.01

Table S4.17. Statistical data comparing the partially oxidized alginate gels with regards to the fraction of modified alginate in the gels. The values for Young's modulus are given as the mean of the gels. Abbreviations: n = number of gels. SD = standard deviation. POA = partially oxidized alginate. βCyD = β-cyclodextrin. POA-MeOTyr = POA grafted with L-Tyrosine methyl ester. POA-βCyD = POA grafted with β-cyclodextrin. POA-GRGDSP = POA grafted with peptide GRGDSP.

Fraction of modified alginate	Gel A / Gel B	Young's modulus (kPa)	SD	n	p-value
0.25	POA 0.08 / POA MeOTyr	29.3 / 21.1	1.3 / 2.4	6 / 10	<0.01
	POA 0.08 / POA βCyD	29.3 / 21.9	1.3 / 4.4	6 / 10	<0.01
	POA MeOTyr / POA βCyD	21.1 / 21.9	2.4 / 4.4	10 / 10	1.0
0.50	POA 0.08 / POA MeOTyr	23.8 / 15.0	2.4 / 2.1	8 / 10	<0.01
	POA 0.08 / POA βCyD	23.8 / 14.5	2.4 / 1.2	8 / 10	<0.01
	POA 0.08 / POA GRGDSP	23.8 / 14.0	2.4 / 0.9	8 / 8	<0.01
	POA MeOTyr / POA βCyD	15.0 / 14.5	2.1 / 1.2	10 / 10	1.0
	POA MeOTyr / POA GRGDSP	15.0 / 14.0	2.1 / 0.9	10 / 8	1.0
	POA βCyD / POA GRGDSP	14.5 / 14.0	1.2 / 0.9	10 / 8	1.0
0.75	POA 0.08 / POA MeOTyr	7.1 / 7.7	5.2 / 0.4	7 / 10	1.0
	POA 0.08 / POA βCyD	7.1 / 6.7	5.2 / 1.2	7 / 9	1.0
	POA MeOTyr / POA βCyD	7.7 / 6.7	0.4 / 1.2	10 / 9	1.0

Table S4.18. Statistical data comparing the partially oxidized alginate gels with regards to the fraction of modified alginate in the gels. The values for syneresis are given as the mean of the gels. Abbreviations: n = number of gels. SD = standard deviation. POA = partially oxidized alginate. βCyD = β-cyclodextrin. POA-MeOTyr = POA grafted with L-Tyrosine methyl ester. POA-βCyD = POA grafted with β-cyclodextrin. POA-GRGDSP = POA grafted with peptide GRGDSP.

Fraction of modified alginate	Gel A / Gel B	Syneresis (%)	SD	n	p-value
0.25	POA 0.08 / POA MeOTyr	23.0 / 25.4	0.6 / 0.5	6 / 10	0.066
	POA 0.08 / POA βCyD	23.0 / 19.5	0.6 / 1.1	6 / 10	<0.01
	POA MeOTyr / POA βCyD	25.4 / 19.5	0.5 / 1.1	10 / 10	<0.01
0.50	POA 0.08 / POA MeOTyr	15.2 / 22.9	0.8 / 2.6	8 / 10	<0.01
	POA 0.08 / POA βCyD	15.2 / 15.2	0.8 / 1.0	8 / 10	1.0
	POA 0.08 / POA GRGDSP	15.2 / 19.0	0.8 / 1.8	8 / 8	<0.01
	POA MeOTyr / POA βCyD	22.9 / 15.2	2.6 / 1.0	10 / 10	<0.01
	POA MeOTyr / POA GRGDSP	22.9 / 19.0	2.6 / 1.8	10 / 8	<0.01
	POA βCyD / POA GRGDSP	15.2 / 19.0	1.0 / 1.8	10 / 8	<0.01
0.75	POA 0.08 / POA MeOTyr	17.7 / 20.9	1.1 / 0.8	3 / 10	0.044
	POA 0.08 / POA βCyD	17.7 / 15.6	1.1 / 3.3	3 / 9	0.50
	POA MeOTyr / POA βCyD	20.9 / 15.6	0.8 / 3.3	10 / 9	<0.01

Table S4.19. Statistical data comparing the partially oxidized alginate gels with regards to the fraction of modified alginate in the gels. The values for deformation at rupture (compression) are given as the mean of the gels. Abbreviations: n = number of gels. SD = standard deviation. POA = partially oxidized alginate. βCyD = β-cyclodextrin. POA-MeOTyr = POA grafted with L-Tyrosine methyl ester. POA-βCyD = POA grafted with β-cyclodextrin. POA-GRGDSP = POA grafted with peptide GRGDSP.

Fraction of modified alginate	Gel A / Gel B	Deformation (%)	SD	n	p-value
0.25	POA 0.08 / POA MeOTyr	57.9 / 58.0	2.4 / 2.3	6 / 10	1.0
	POA 0.08 / POA βCyD	57.9 / 61.3	2.4 / 7.1	6 / 10	0.86
	POA MeOTyr / POA βCyD	58.0 / 61.3	2.3 / 7.1	10 / 10	0.76
0.50	POA 0.08 / POA MeOTyr	53.9 / 61.6	3.1 / 6.8	7 / 10	<0.01
	POA 0.08 / POA βCyD	53.9 / 24.0	3.1 / 1.8	7 / 10	1.0
	POA 0.08 / POA GRGDSP	53.9 / 58.0	3.1 / 2.1	7 / 8	0.65
	POA MeOTyr / POA βCyD	61.6 / 24.0	6.8 / 1.8	10 / 10	<0.01
	POA MeOTyr / POA GRGDSP	61.6 / 58.0	6.8 / 2.1	10 / 8	0.74
	POA βCyD / POA GRGDSP	24.0 / 58.0	1.8 / 2.1	10 / 8	0.54
0.75	POA 0.08 / POA MeOTyr	53.3 / 58.3	3.9 / 2.5	7 / 10	0.28
	POA 0.08 / POA βCyD	53.3 / 48.6	3.9 / 3.3	7 / 9	0.38
	POA MeOTyr / POA βCyD	58.3 / 48.6	2.5 / 3.3	10 / 9	<0.01

Table S4.20. Statistical data comparing the partially oxidized alginate gels with regards to the fraction of modified alginate in the gels. The values for stress at rupture are given as the mean of the gels. Abbreviations: n = number of gels. SD = standard deviation. POA = partially oxidized alginate. βCyD = β-cyclodextrin. POA-MeOTyr = POA grafted with L-Tyrosine methyl ester. POA-βCyD = POA grafted with β-cyclodextrin. POA-GRGDSP = POA grafted with peptide GRGDSP.

Fraction of modified alginate	Gel A / Gel B	Stress at rupture (N)	SD	n	p-value
0.25	POA 0.08 / POA MeOTyr	21.3 / 15.8	3.2 / 3.6	6 / 10	0.021
	POA 0.08 / POA βCyD	21.3 / 24.0	3.2 / 2.0	6 / 10	0.80
	POA MeOTyr / POA βCyD	15.8 / 24.0	3.6 / 2.0	10 / 10	<0.01
0.50	POA 0.08 / POA MeOTyr	11.3 / 9.9	3.3 / 4.6	7 / 10	1.0
	POA 0.08 / POA βCyD	11.3 / 10.9	3.3 / 1.2	7 / 10	1.0
	POA 0.08 / POA GRGDSP	11.3 / 13.0	3.3 / 1.8	7 / 8	0.99
	POA MeOTyr / POA βCyD	9.9 / 10.9	4.6 / 1.2	10 / 10	1.0
	POA MeOTyr / POA GRGDSP	9.9 / 13.0	4.6 / 1.8	10 / 8	0.52
	POA βCyD / POA GRGDSP	10.9 / 13.0	1.2 / 1.8	10 / 8	0.93
0.75	POA 0.08 / POA MeOTyr	7.1 / 6.9	1.7 / 1.9	7 / 10	1.0
	POA 0.08 / POA βCyD	7.1 / 2.8	1.7 / 0.4	7 / 9	0.14
	POA MeOTyr / POA βCyD	6.9 / 2.8	1.9 / 0.4	10 / 9	0.091

Table S4.21. Statistical data comparing the gels with epimerized POA-MeOTyr with gels containing non-epimerized POA-MeOTyr (W_{POA*} =0.50). The values are given as the mean and standard deviation of the data for the epimerized gels. Abbreviations: n = number of gels. POA-MeOTyr = POA grafted with L-Tyrosine methyl ester.

Young's modulus (kPa)	p-value	Syneresis (%)	p-value	Deformation at rupture (%)	p-value	Stress at rupture (kg)	p-value
12.9 ± 3.5, n = 6	0.21	21.7 ± 1.7, n = 6	0.27	53.3 ± 1.9, n = 6	0.0039	1.2 ± 0.4, n = 6	0.54

Statistical data for the modified alginate gels are here given with regards to the Young's modulus, syneresis and compression and stress at rupture.
